# Nature’s carriers: leveraging extracellular vesicles for targeted drug delivery

**DOI:** 10.1080/10717544.2024.2361165

**Published:** 2024-06-04

**Authors:** Qi Chen, Yuyi Zheng, Xuhong Jiang, Yi Wang, Zhong Chen, Di Wu

**Affiliations:** a Interdisciplinary Institute for Medical Engineering, Fuzhou University, Fuzhou, P. R. China; b Key Laboratory of Neuropharmacology and Translational Medicine of Zhejiang Province, School of Pharmaceutical Sciences, Zhejiang Chinese Medical University, Hangzhou, China; c Epilepsy Center, Department of Neurology, The First Affiliated Hospital of Zhejiang Chinese Medical University, Hangzhou, PR China; d Zhejiang Rehabilitation Medical Center, The Third Affiliated Hospital of Zhejiang, Chinese Medical University, Hangzhou, PR China

**Keywords:** Extracellular vesicles, drug delivery system, nanoparticle, carrier

## Abstract

With the rapid development of drug delivery systems, extracellular vesicles (EVs) have emerged as promising stars for improving targeting abilities and realizing effective delivery. Numerous studies have shown when compared to conventional strategies in targeted drug delivery (TDD), EVs-based strategies have several distinguished advantages besides targeting, such as participating in cell-to-cell communications and immune response, showing high biocompatibility and stability, penetrating through biological barriers, etc. In this review, we mainly focus on the mass production of EVs including the challenges and strategies for scaling up EVs production in a cost-effective and reproducible manner, the loading and active targeting methods, and examples of EVs as vehicles for TDD in consideration of potential safety and regulatory issues associated. We also conclude and discuss the rigor and reproducibility of EVs production, the current research status of the application of EVs-based strategies to targeted drug delivery, clinical conversion prospects, and existing chances and challenges.

## 1. Introduction

**Figure 1.**
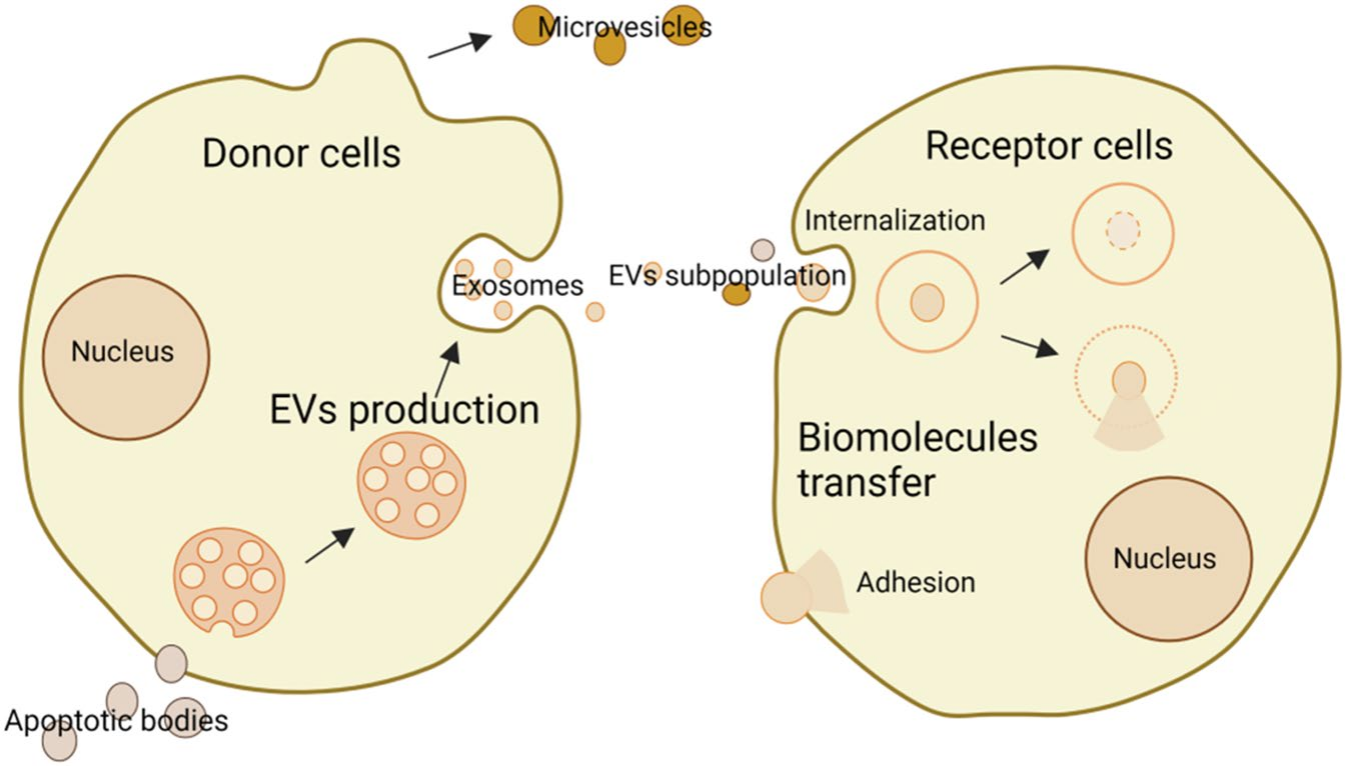
Schematic illustration of the production of EVs from donor cells and the transfer to receptor cells via internalization or adhesion (drawn by BioRender app).

Extracellular vesicles (EVs), which include a diverse range of subtypes, such as exosomes, microvesicles, and apoptotic bodies, are secreted by donor cells to receptor cells for transferring biomolecules and exchanging information (Wang et al., 2023). They can either adhere to and/or be internalized to receptor cells. They are small phospholipid membrane-enclosed entities without the capabilities to replicate, presenting in almost all biofluids, including blood, saliva, urine, and so on (Cheng & Hill, 2022). Their uneven sizes are from 20 nm to 10 μm or more (O’Brien et al., 2020). The compositions of EVs are heterogeneous and relevant mechanisms of action are different, thus it is hard to distinguish the categories of EVs. The details are shown in Figure 1. To understand and use EVs better, more studies are still needed on many unknowns. Previously, EVs were known as metabolism wastes. But now, they are also considered drug delivery masters.

Drug delivery systems deliver drugs in a controlled pattern which improves the efficacy of conventional drugs and overcomes the expensive and time-consuming of new drug development (Gao et al., 2023). Particularly, lipid-based nanocarriers have led to clinical translation. At the same time, the field of EVs which are natural lipid bilayer-enclosed vesicles has grown rapidly and attracted considerable interest (Elsharkasy et al., 2020). Due to the characteristics of EVs that come from their originating cells, for example, EVs from cancer cells show homologous targeting behaviors, which can be used to make cell membrane-coated nanoparticles for targeted drug delivery to the same cancers. In recent years, in addition to direct uses of EVs, there are various methods to engineer them to retain or enforce favorable qualities and to overrule or mask unfavorable qualities (de Jong et al., 2019). These have expanded the functions and applications of EVs, and meanwhile, provide references to the evaluation of natural EVs and reproducible delivery.

Here, to understand the roles of EVs in-depth, we summarize the current state of EVs-based strategies in targeted drug delivery, the pros and cons related to their translation and critically discuss their prospects in next-generation therapeutics.

## 2. Mass production of extracellular vesicles

EVs-based strategies, as novel and expanding fields, have great potential in the translation to clinical practices. In the field of targeted drug delivery, small sizes and homologous sources of EVs may allow them to cross the barriers (Wang et al., 2023). Moreover, on one hand, EVs contain many receptors and proteins on their membranes that can interact with specific microenvironment and achieve precisely targeted delivery by improving the targeting and specificity of drugs and reducing the side effects of drugs on nonspecific cells. On the other hand, the membrane composition of EVs is equivalent to the membrane composition of the targeting cells, which can effectively protect the original characteristics of drugs from undergoing any excessive changes, and overcome the problems of premature clearance by the immune system, liver, and other degradation organs before the drugs take effect. However, the productivity of EVs is low and becomes the major limitation for clinical applications. Therefore, mass production of EVs is highly demanded, providing more high-quality raw materials to develop drug carriers, which can not only improve the level of medical technology but also open up new paths and prospects for drug development and treatment.

To date, there have been various separation, purification, and enrichment methods for collecting EVs, such as ultracentrifugation (Roerig et al., 2022), ultrafiltration (Ko et al., 2023), size exclusion chromatography (Lisi et al., 2023), polyethylene glycol precipitation (Jalaludin et al., 2023) and so on. Ultracentrifugation separates EVs based on the density, size, and shape of solutes. Although ultracentrifugation is thought to be the gold standard to get EVs for research purposes, its scalability is unsatisfactory (Royo et al., 2020). The equipment which is cumbersome costs high. What’s more, high-speed centrifugation and long running time may damage EVs. Ultrafiltration uses ultrafiltration tubes bearing centrifugal filters. The choice of ultrafiltration tubes affects the yield of EVs, which can vary. Size exclusion chromatography costs low and manipulates fast. However, it is easy to induce deterioration and loss of EVs due to attaching to the stationary phase. Polyethylene glycol precipitation is indeed easily scalable, but it precipitates more than just EVs from the culture medium, resulting in a less pure preparation. Ultrafiltration, size exclusion chromatography, and polyethylene glycol precipitation are comparatively easy to translate to large-scale EV manufacturing. However, it still remains challenges, for instance, how to reduce the costs.

**Figure 2.**
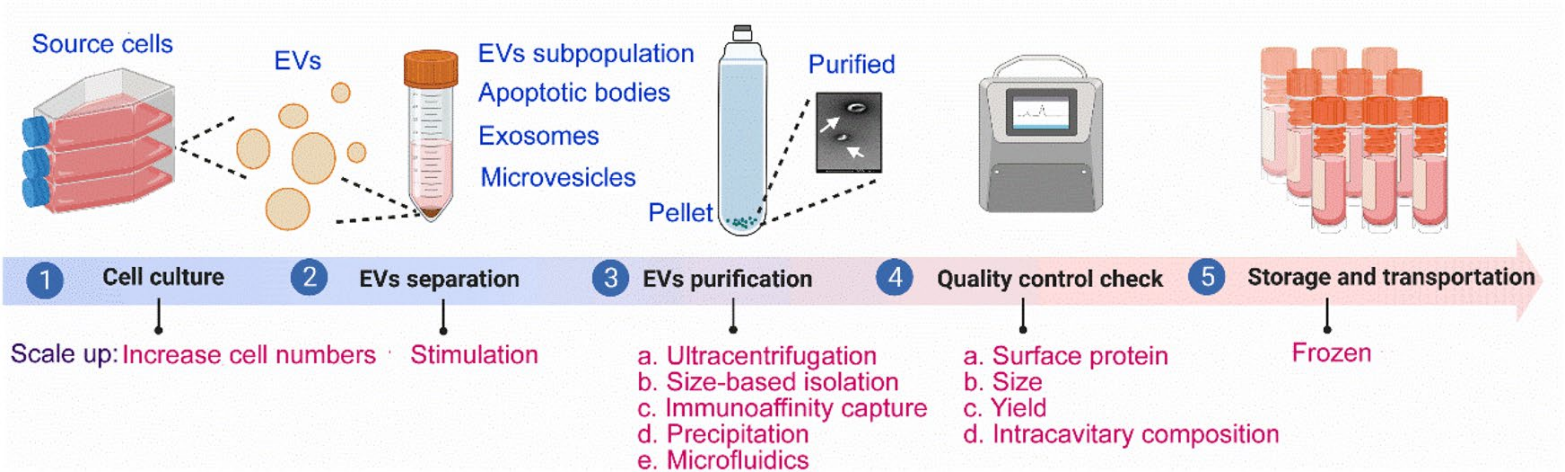
Flowchart of the production of EVs and related scale-up points (drawn by BioRender app).

To scale up, we can get involved in some steps during the production of EVs to boost the yield (Figure 2). The main steps for the production of EVs include cell culture, separation, purification, quality control check, storage, and transportation. At the stage of cell culture, the production of EVs tends to require massive cellular culture systems (Popowski et al., 2020). By increasing the surface area of the culture and minimizing the volume of media, EVs could be concentrated. For instance, the types of flasks and bioreactors using for collecting EVs have hyperflasks, roller bottles, perfusion, fixed bed or spinner flasks. Besides, cell lines, cell-culture conditions, such as mild temperature, pH, nutrient and oxygen levels, cell-culture time, stimulation methods, etc. affect the yield and quality of EVs (Witwer et al., 2017). Suspension cells offer a more convenient and scalable EVs production system compared to adherent cells such as HEK-293T, primarily because of their more straightforward propagation procedures (Zheng et al., 2022). As for the effects of different storage temperatures and storage buffers, the field lacks defined and standardized conditions for EV preservation. So far, there have been a number of studies investigating the storage conditions, among them, phosphate-buffered saline has been the preferred storage buffer and recommended to store at −80 °C (Görgens et al., 2022). It’s worth mentioning that some stresses (e.g. oxidative stress, heat shock, etc.) can increase the secretion of EVs (Mateescu et al., 2017). When cells are exposed to stressful conditions, they release more EVs to advertise and deliver stress-response messages. Next, we will discuss the cell stress technologies for increasing EVs production. Specifically, electrical stimulation, pharmacologic agents, gamma rays, x-rays, ultraviolet light, visible light, sound waves, shear stress, cell starvation, alcohol, pH, and genetic manipulation are often utilized (Erwin et al., 2023). For example, an applied electric field of less than 5 mV/mm, with frequencies ranging from 2 to 200 Hz, results in increased concentration and miRNA cargo of EVs derived from astrocytes (Wang et al., 2019). Treatment of MDA-MB-231, HeLa, and MCF7 cells with the drug homosalate could increase their EVs production (Grisard et al., 2022). Mutschelkanus et al. discovered that the secretion of EVs in head and neck cancer cell lines (BHY and FadU) increased by 6 to 7 times after exposure to gamma radiation doses ranging from 3 to 6 Gy (Mutschelknaus et al., 2016). After being treated with x-rays at a rate of 2.3 Gy/min and a dose of 4 Gy, glioblastoma cells, stem-like cells, and astrocytes exhibited an increase in their rates of EV secretion. Glioblastoma cells showed a 1.23- to 1.79-fold increase, stem-like cells showed a 2.6-fold increase, and astrocytes showed a 1.71-fold increase (Arscott, et al. 2013). However, it is important to note that cell stress technologies have the potential to alter the characteristics of EVs. Therefore, in order to fully utilize them, it is essential to address the challenge of elucidating the differences between various cell stress technologies. Hence, according to the fact, choosing parent cells that are prone to the high productivity of EVs, optimizing the cell culture conditions, utilizing stimulants, etc. could realize the goal of mass production.

## 3. Drug loading and active targeting methods

**Figure 3.**
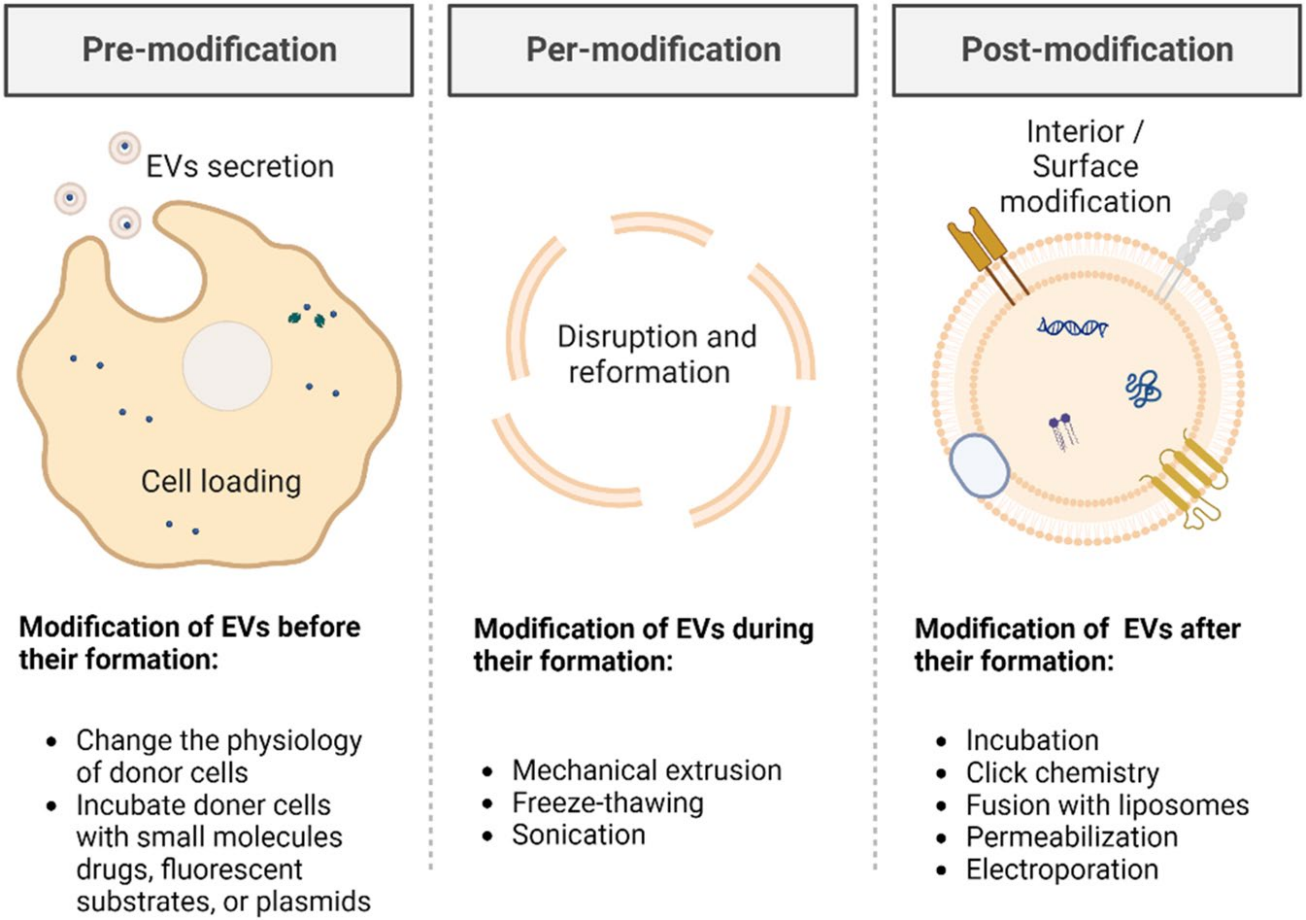
Schematic of drug loading and active targeting methods for EVs-based strategies (drawn by BioRender app).

When employing EVs as a drug delivery system, their structure can be regarded as a hydrophilic lumen, encapsulated in a protein-lipid membrane, which contributes to the encapsulation of hydrophobic, hydrophilic, and amphiphilic molecules (van der Koog et al., 2022). In addition to the direct use of natural EVs, artificially modified and/or synthesized EVs have also inspired the development. Next, we will introduce three different strategies (pre-modification, per-modification, or post-modification) (Figure 3) for drug loading and active targeting in several cases.

### 3.1. Pre-modification

Pre-modification refers to the modification of EVs before their formation, which is a straightforward method. Desired modification substances, mainly small molecules drugs, fluorescent substrates, or plasmids, which can cross the donor cell membrane, can be incorporated into source cells for developing EVs. By using this approach, EVs possess the desired modifications before being released into the external environment.

It has been reported in the literature that RAW264.7 macrophages were first transfected with murine IL-10 plasmid and then stimulated with dexamethasone for developing IL-10-expressed EVs which can be further used in treating acute kidney injury (AKI) (Tang et al., 2020). Liang et al. constructed engineered donor 293 T cells that expressed target-Her2-LAMP2-GFP and generated target-specific EVs to efficiently target well-established 5-Fluorouracil-resistant colon cancer cells HCT-116 (Liang et al., 2020). Nie et al. polarized macrophages into anti-tumoral M1 macrophages and modified them with azide groups to produce azide-functionalized M1 EVs, which can further react with the dibenzocyclooctynes (DBCO)-modified pH-sensitive antigens, anti-CD47 antibody (aCD47) and anti-signal regulatory protein alpha antibody (aSIRPa) (Nie et al., 2020). Pinto et al. added meta(tetrahydroxyphenyl)-chlorin (mTHPC) into the media of mesenchymal stem/stromal cells (MSCs) for overnight incubation, followed by several steps to remove free drugs, eliminate cell debris and separate EVs-contained mTHPC (EVs-mTHPC) (Pinto et al., 2021). EVs-mTHPC showed enhanced tumoral selectivity when compared to free mTHPC or liposomal mTHPC formulation. Another study found EVs from CD47-overexpressed plasmid-transfected 293 T cells can prevent the clearance by the phagocytosis of the mononuclear phagocyte system (MPS), and thus accumulate more in tumor tissues (Du et al., 2021).

In these cases, the modification substances are crucial to the donor cells. They first change the physiology of donor cells and then indirectly influence the EVs production. Therefore, states of the donor cells, such as vitality, differentiation, apoptosis, etc., as well as the proportion and isolation of successfully modified cells, need to be fully considered when using pre-modification. For instance, if the donor cells have low vitality or exhibit significant apoptosis, it may influence the success rate of modification and the functional performance of the modified cells. If the modified cells cannot be efficiently separated from the unmodified cells, it may impact the accuracy and feasibility of experiments or therapies.

Thus, it is important to weigh the potential drawbacks against the advancements that pre-modification of cells can bring. Modifying cells at the genetic level can sometimes result in unintended changes elsewhere in the genome, leading to off-target effects. These off-target effects can have unpredictable consequences on the behavior and function of the modified cells. Also, there may be safety concerns associated with pre-modified cells. The introduced genetic changes could potentially lead to adverse effects, such as the development of tumors or unwanted immune responses. Moreover, the use of pre-modified cells raises ethical considerations, particularly when it comes to gene editing technology like CRISPR. The manipulation of genetic material raises questions about the potential consequences and long-term implications of altering the fundamental building blocks of life. To sum up, off-target effects, safety concerns, ethical considerations, and so on should be taken into account when utilizing pre-modification.

### 3.2. Per-modification

Per-modification involves modifying EVs during their formation process, which is a relatively facile physical method. It could be viewed as a method to disrupt the structure of EVs and reform their components. To be noted, from previously published studies, methods of per-modification and methods of post-modification have partial overlaps.

Mechanical extrusion is a physical method that can be used to disrupt EVs and release the biomolecules inside them. The process typically involves physical squeezing and shearing forces, which can shape EVs. Zha et al. reported biotin-functionalized 1,2-dioleoyl-sn-glycero-3-phosphoethanol­aminepoly (ethylene glycol) (DSPE-PEG) (DSPE-PEG-Biotin) modified ATDC5, a mouse chondrogenic progenitor cell line, after serially extruded through 7 μm, 5 μm, and 0.22 μm filters, could generate EVs with biotin (Zha et al., 2020). Wang et al. used mechanical extrusion to fabricate EVs loaded with various drugs (gelonin, paclitaxel, and siRNA) (Wang et al., 2023). The average loading efficiencies of these drugs were 31.2 ± 2.8%, 27.4 ± 2.3%, and 3.7 ± 0.4%, respectively.

Freeze-thawing exploits the volume change of water during repeatedly freezing and thawing to rupture and reshape EVs. Haney et al. loaded a potent antioxidant, catalase, into Raw 264.7 EVs by freezing at −80 °C and thawing at room temperature for three cycles (Haney et al., 2015). Sato et al. fused Raw264.7 cell-derived EVs with PEG-modified liposomes by the freeze-thaw method (Sato et al., 2016). The resulting PEG-modified EVs increased the circulation time in blood. They pointed out that the technique should also be useful for encasing therapeutic agents. Wu et al. displayed folic acid on the surface of mRNA-encapsulated liposomes and then developed a lipid-EV hybrid system through the freeze-thaw method. Briefly, the liposomes and EVs were mixed in equal ratios. The mixture was put at −80 °C for 15 min and then at 37 °C for 15 min, lasting three cycles (Wu et al., 2023).

Sonication introduces ultrasonic energy to generate shear force and pressure, which helps to open the structure of cells and EVs. The parameters and procedures for sonication may vary depending on the device and the source of EVs. Go et al. resuspended isolated EVs from human U937 monocytes in the absence or presence of different concentrations of dexamethasone, and subjected them to sonication under a UP400S sonifier (Go et al., 2019). Through this method, they got EV-mimetic ghost nanovesicles, which realized the targeting to activated endothelial cells, reduced the release of IL-8, and mitigated systemic inflammatory response syndrome. Yerneni et al. sonicated the mixture of curcumin, albumin and EVs to load albumin and curcumin into EVs (CA-EVs) (Yerneni et al., 2022). The loading was done sequentially using mild sonication parameters, six cycles for 3 minutes, 30 s on/off, cooling period between each cycle for 2 minutes, to load albumin first, followed by curcumin.

In addition to mechanical extrusion, freeze-thawing, and sonication, there are other per-modification that can be used to disrupt EVs and reconstitute them. The choice of which method to use depends on the purpose of the experiment, the nature of the EVs, and the desired processing effect. Also note that per-modification has certain drawbacks, such as relatively low feasibility and efficiency. The process of per-modification, depending on the specific techniques employed, can potentially disrupt cellular homeostasis, and cellular functions, or even induce cellular stress responses, leading to potential deviations from expectations. It is crucial to carefully assess and optimize per-modification methods to minimize potential cellular damage and ensure that the modifications introduced do not compromise the biological properties and functionalities of the EVs.

### 3.3. Post-modification

Post-modification refers to modifying EVs after their formation, which is a flexible method. Post-modification methods typically include physical or chemical approaches to stably interact the modification substances with EVs.

Incubation is a commonly used method for post-modification. Incubation-induced spontaneous fusion occurs as substrates for incubation have similar structures, for example, lipid bilayers. Kooijmans et al. mixed EVs derived from Neuro2A cells or platelets with PEGylated micelles and incubated them at 40 °C, indicative of a post-insertion mechanism (Kooijmans et al., 2016). This method is a promising tool that allows for the extended application of clinically approved antibodies or many well-studied peptides. Zhang et al. prepared a P-selectin binding peptide (PBP, CDAEWVDVS) to conjugate phospholipid derivative, which could be inserted into EVs through incubation for 30 min (Zhang et al., 2023). They proved the modification efficiency increased along with incubation time and temperature and the PBP-engineered EVs (PBP-EVs) showed P-selectin targeting ability.

Click chemistry, which can be an ideal method for membrane functionalization, is widely used in surface loading and surface modification. Xu et al. mixed EVs suspension with dibenzylcyclooctyne-NHS ester (DBCO-NHS) and then added Alexa FluorTM 488 azide (AF488-azide), fabricating fluorescence labeled EVs through copper-free click chemistry-based labeling (Xu et al., 2020). Ruan et al. reported that DBCO-PEG_4_-NHS reacted with EVs from M2 microglia cells to prepare EVs-DBCO (Ruan et al., 2023). Next, EVs-DBCO were further reacted with azide-labeled SDF-1 and azide-labeled DA7R to construct functionalized EVs, which at the injured site, can recruit neural stem cells (NSCs) and improve their differentiation. Lee et al. collected EVs from metabolic glycoengineering human mesenchymal stem cells (hMSCs) which has azides on the surface and loaded smoothened agonists (SAG) into the core (Lee et al., 2023). A bone-targeting ligand, named alendronate (ALD), was functionalized on the surface of ALD-loaded EVs through the click reaction between dibenzocyclooctyne (DBCO) and azide. This post-modification EVs increased the osteogenic capacity through the improvement of targeting behavior and drug efficacy.

Fusion with liposomes gives rise to many new hybrid drug delivery systems. EV-liposome hybrids are expected to benefit from both the liposome properties, such as high encapsulation efficiency and high loading capacity, and natural EV properties, such as potential cell-type targeting. Fusion methods are various, including the above Section 3.2 mentioned physical methods. When choosing the fusion method, operability, fusion efficiency, biological activity, etc. should be taken into consideration. For example, the efficacy of hyperthermic intraperitoneal chemotherapy (HIPEC) is restricted by drug penetration and drug resistance. To overcome the challenges, Lv et al. produced ‘self-protein’ (CD47) and/or granulocyte-macrophage colony-stimulating factor (GM-CSF) expressed EVs from genetically engineered fibroblasts, which were then fused with docetaxel (DTX)-loaded thermosensitive liposomes (Lv et al., 2020). The systems preferentially accumulated in metastatic peritoneal cancer and released payloads to inhibit tumor development. When HIPEC is co-administered, the efficacy was enhanced. Evers et al. evaluated EV-liposome hybrid nanoparticles (hybrids), liposomes and EVs, and found hybrids combine properties of both liposomes and EVs, showing alteration in terms of functional behaviors (Evers et al., 2022). They produced hybrids that functionally deliver RNA by lipid-film hydration followed by extrusion. They also observed that higher siRNA-AF647 signal along with an increase in the number of EVs in the formulation. In addition, they observed the fluorescence signal from liposome in corresponds to siRNA, which indicates the EV hybrids carrying lipids while simultaneously complexing siRNA.

Permeabilization, using chemical reagents such as saponin, triton and so on, permeabilizes cell membranes and separates biomembrane components. For example, Fuhrmann et al. mentioned in a comparative study, saponin-assisted (SP) drug loading for EVs didn’t compromise the drug delivery abilities of EVs in the aspects of constitution and functionality but enhanced the drug loading (Fuhrmann et al., 2015). The facile method was through incubating EVs and drugs with saponin at room temperature. Saponin is a surfactant molecule, which can form complexes with cholesterol in EVs. With the help of saponin, drug loading into EVs increased 11-fold when compared to the passive loading without saponin. Wang et al. loaded EVs with tetraacetylated N-azidoacetyl-D-mannosamine (Ac4ManNAz) by the reversible permeabilization method (Wang et al., 2017). They used activated streptolysin O (SLO), a pore-forming bacterial toxin, as a reversible membrane permeabilization tool to open the structure of EVs.

Electroporation is the most commonly used method to incorporate siRNA or miRNA into EVs (Kar et al., 2023). It involves a high-intensity electric field for causing instantaneous changes in the permeability of EVs. Molecules ready for modification could enter the pores created in the EVs during electroporation, while the EVs are restored after loading. Kooijmans et al. used an electroporation method (post-modification method) to prepare EVs with fluorescently labeled siRNA (Kooijmans et al., 2013). They studied and optimized the electroporation process of siRNA. From their results, siRNA aggregated could result in high encapsulation efficiency wrongly. Pomatto et al. showed successful miRNA (miR-31 and miR-451a) loading into plasma EVs by electroporation for promoting apoptosis of cancer cells and silencing target genes (Pomatto et al., 2019). In this study, they not only optimized the electroporation protocol but also evaluated the functional delivery of anti-tumor miR-31 and miR-451a.

From this section, we could find that post-modification, when compared to pre-modification and per-modification, makes EV more prone to loss or degradation during processing because of the additional steps and techniques to extract original EVs. This complexity and time requirement could add limitations to experimental or clinical applications.

In sum, these modification methods can be combined according to specific needs to achieve the desired modification effects on EVs. Different modification methods are applicable to various research purposes and application requirements, and the most suitable method or strategy can be chosen based on specific circumstances. In addition, it is worth noting that, there may be immunogenicity concerns when modifying EVs in unnatural ways. When modified with foreign or synthetic materials, EVs can trigger an immune response in the recipient. The immune system may recognize these modified EVs as foreign or non-self, leading to the production of antibodies or activation of immune cells. This immune response can impact the effectiveness and safety of modified EVs in therapeutic applications. Therefore, careful consideration and extensive evaluation of the immunogenicity profile are essential when modifying EVs in unnatural ways. To mitigate unwanted immune response, we could try to use homologous EVs. We need to balance the drawbacks and benefits of different drug loading and active targeting methods, in consideration of practical applications and research objectives. Furthermore, with ongoing technological advancements and scientific progress, we can expect further improvements in our understanding and control of EVs modifications, potentially overcoming some of the current limitations.

## 4. Extracellular vesicles as vehicles for targeted drug delivery

Diverse examples validate the successful use of EVs, some of which are described later in this review. We aim to report overviews on topics of drug models, disease models and delivery routes in this section for a deeper comprehension of the features of EVs-based drug delivery systems and their application-specific functional activities.

### 4.1. Drug models

It has been proposed that EVs may have the ability to serve as drug delivery systems for a variety of therapeutic medicines in recent research, including chemical molecules, nucleic acids, proteins, and etc. (Table 1).

**Table 1.** Targeted delivery of different drugs using extracellular vesicles.

Source	Cargo	Encapsulation efficiency	Reference
Bone marrow MSCs (BMSCs)	DOX	12%,77.9 ± 0.5%, ∼80%	(Wei et al., 2019; Liu et al., 2020; Li et al., 2022; Wang et al., 2022)
MSCs	DOX	∼90%, 34.2%, 35%	(Bagheri et al., 2020; Liu et al., 2020; Yang et al., 2022)
Immature dendritic cells	DOX	20%	(Tian et al., 2014)
HEK 293T cells	DOX	11.73%	(Wang et al., 2022)
Macrophages	DOX	82% ∼ 99%	(Rayamajhi et al., 2019)
Neutrophil	DOX	13.71 ± 0.7%	(Wang et al., 2021)
M1-macrophages	PTX	19.55 ± 2.48%	(Wang et al., 2019)
Macrophages	PTX	28.29 ± 1.38%, 33%	(Kim et al., 2016; 2018)
MDA-MB-231 cell	PTX	87.6%	(Wang et al., 2020)
Human monocyte cells	miRNA 159	5.33%	(Gong et al., 2019)
Hypoxic SKOV3 cells	miRNA-181c-5p	–	(Yang et al., 2022)
Murine B cell	miRNA-155 mimic and miRNA-155	55.06%	(Momen-Heravi et al., 2014)
Chondrocytes	miRNA-140	60%	(Liang et al., 2020)
HEK 293T cell	siRNA	80%	(Zheng et al., 2019)
Autologous breast cancer cells	siS100A4	86.70 ± 1.22%	(Zhao et al., 2020)
Raw 264.7	Catalase	18.5 ± 1.3 %	(Haney et al., 2020)
HEK 293T cell	Lysosome-associated membrane glycoprotein 2b	80% ∼ 90%	(Wang et al., 2018)
SKBR-3, SNU-216 and MCF-7 cell	Trastuzumab emtansine	75%	(Barok et al., 2018)
Dendritic cell	α-fetoprotein	–	(Lu et al., 2017)

Inefficient targeting and side effects have made it difficult to use conventional chemotherapeutic drugs. Extensive research demonstrated that using EVs as a tailored delivery system can enhance drug accumulation in disease tissue, improve therapeutic efficacy, and lessen systemic toxicity (Tian et al., 2014; Saari et al., 2015; Zhang et al., 2020; Xu et al., 2022). To cite an example, although doxorubicin (DOX) is a traditional chemotherapeutic model drug used to treat different types of cancers, its use in clinical practice has been constrained due to high toxicity. Thus, researchers have employed MSC-derived EVs to boost the uptake of DOX and increase its anti-tumor effects while lowering its toxicity to other organs by taking advantage of MSCs’ preference for tumor tissue (Liu et al., 2020; Li et al., 2022; Tian et al., 2022; Wei et al., 2022; Zhang et al., 2022). What’s more, the anticancer effects of DOX-loaded EVs which have EVs from other sources, including macrophages, dendritic cells, HEK 293T cells, and erythrocytes, are superior to those of free DOX (Schindler et al., 2019; Zhang et al., 2020). Paclitaxel (PTX), due to its poor solubility in water, is often dissolved in methanol or ethanol. Nevertheless, organic solvents can exacerbate the side effects of PTX, such as allergic reactions and reactions involving the cardiovascular system (Zhu et al., 2019). Additionally, patients often get resistant to PTX as the treatment duration lengthens, which reduces its efficacy. To solve this problem, Wang et al. created a drug delivery system using PTX loaded in EVs from M1 polarized macrophage and discovered that toxicity of PTX against drug-resistant cells was boosted by over 50 times (Wang et al., 2019; 2022). Moreover, apart from lung cancer, such delivery systems showed effective targeting and anticancer effects in breast cancer and pancreatic cancer.

Nucleic acid drugs, which encompass DNA and RNA molecules had been investigated as potential medicines for the treatment of protein-related diseases by modulating the expression of target proteins. In this context, microRNA (miRNA) is an RNA molecule that is commonly present in eukaryotes and has a length of around 18 to 22 nucleotides. It has the ability to control gene expression through a number of routes. Extracellular miRNAs are crucial for the detection and management of malignancies (Shahabipour et al., 2016; Lu et al., 2018). For instance, Wang et al. discovered that the delivery of anti-miR-214 through EVs could potentially overcome cisplatin resistance, leading to accelerated tumor cell apoptosis and consequently reducing tumor progression (Wang et al., 2018). In another study, glioma-bearing rats showed significant improvement in treatment efficacy when treated with miR-214-loaded EVs (Lang et al., 2018). Through loading various miRNAs, miRNA-loaded tumor-derived EVs can be released into the tumor microenvironment and transferred to macrophages to further realize various miRNA effects (Li et al., 2022; Njock et al., 2022; Yang et al., 2022; Wang et al., 2023). Small interfering RNAs (siRNAs), which are crucial for controlling gene expression and disease therapy, are RNA interference initiators that trigger the silence of target mRNAs that are complementary to them. The therapeutic efficacy is unsatisfactory. There are various reasons, for instance, due to their short half-life, immunogenicity, failure to pass the blood-brain barrier, off-target effects and so on (Castanotto & Rossi, 2009; Ciccone et al., 2023). To improve targeting ability and good biocompatibility, EVs from breast cancer cells were used to load sis100A4 (Zhao et al., 2020; Pan et al., 2023). A further investigation by Alvarez-Erviti et al. revealed that the therapeutic potential of EVs for delivering siRNAs in Alzheimer’s disease (AD). They loaded siRNAs into dendritic cell-derived EVs which were prefused with neuron-targeting peptides and found these nanoparticles displayed good brain targeting capabilities (Alvarez-Erviti et al., 2011). In addition, the off-target effect was reduced by blocking the mononuclear macrophage system and enhancing the microvascular permeability of the target tissue.

Therefore, an appropriate drug model is important for EV-based delivery carriers. It helps to evaluate the efficacy of EV-based delivery carriers, such as stability, controlled release properties, and so on, which gives hints to us about how to optimize EV-based delivery carriers.

### 4.2. Disease models

EVs are believed to be Trojan Horses for intractable diseases because they are crucial to intercellular communication and participate in many physiological and pathological processes (Alvarez-Erviti et al., 2011; Njock et al., 2022; GUO et al., 2022), involving participation in activities including angiogenesis, coagulation, cell survival, neuroinflammation, etc. (Yuana et al., 2013) (Table 2).

**Table 2.** Examples of extracellular vesicle-based drug delivery for disease treatment.

Source	Disease	Effect	Reference
Dendritic cells	Cancer	The dendritic cell-EVs present superior anti-tumor effects, and prevent tumor metastasis in mice.	(Li et al., 2023)
B16BL6 cells	Cancer	DC2.4 cells can be significantly activated by B16BL6 cells-EVs.	(Morishita et al., 2016)
Dendritic cells	Degenerative alveolar bone disease	DC EVs encapsulate immunomodulatory cargo from proteolytic degradation, promote T-regulatory cell recruitment, and reduce osteoclast bone loss.	(Elashiry et al., 2020)
Human non-small-cell lung cancer cells	Lung cancer	Human non-small-cell lung cancer cells EVs can efficiently promote the proliferation of CD4+ cells and restrain tumor development.	(Li et al., 2013)
M2 macrophage	Lungade carcinoma	M2 macrophage-derived EVs can enhance cell uptake and promote cell migration, invasion, and angiogenesis.	(Wei et al., 2022)
M2 microglia	Ischemic stroke	EVs from M2 microglia control glial scar formation and inhibit the proliferation of glial cells in rat.	(Li et al., 2021)
M2 microglia	Cerebral ischemia	EVs from M2 microglia promote cerebral white matter regeneration to lessen ischemic injury in mice.	(Li et al., 2022)
Human neural progenitor cells	Cerebral ischemia	Neural progenitor cells-derived EVs show a strong inhibitory effect on the inflammatory response.	(Tian et al., 2021)
MSCs	Ischemia-reperfusion acute kidney injury	MSC-EV alleviates oxidative damage by activating Keap1-Nrf2 signaling.	(Zhou et al., 2023)
Plasma	Cerebral ischemia	Ischemic preconditioning-EVs can improve N2A cell viability by reducing the production of small G protein, blocking downstream pathways.	(Li et al., 2021)
Adipose-derived stem cells	Ischemic stroke	By promoting the transition of glial cells from M1 to M2, ADSC-EVs lessen neuronal damage and enhance cognitive function.	(Yang et al., 2022)
MSCs	Cerebral ischemia	MSCs-EVs can effectively target the ischemic area and increase BDNF expression to repair damaged neurons.	(Zhou et al., 2023)
SH-SY5Y cells	PD	EVs from SH-SY5Y cells can be effective in enhancing the transfer of α -synaptic nucleoprotein.	(Alvarez-Erviti et al., 2011)
Raw 264.7	PD	Monocyte- and macrophage-derived EVs can increase drug concentration in the brain and enhance drug efficacy.	(Haney et al., 2015)
Umbilical cord blood mononuclear cells	PD	EVs-supported microRNA (miR)-124-3p can improve drug delivery to the diseased brain area, and improve the therapeutic effect in a mouse model of PD.	(Esteves et al., 2022)
Murine dendritic cells	PD	RVG-overexpressed EVs effectively improve drug delivery and alleviate dopaminergic neuron loss.	(Izco et al., 2019)
Blood	PD	Blood EVs improve the distribution of dopamine in the brain while reducing toxicity to other sites.	(Qu et al., 2018)
BM-MSCs and HEK-293T cells	AD	These EVs enable precise targeting of specific cells for precision therapy.	(Jahangard et al., 2020)
Self-derived dendritic cells	AD	RVG-targeted EVs deliver siRNAs to knock down neuron-specific genes in the brain.	(Alvarez-Erviti et al., 2011)
HEK 293T cells	Atherosclerosis	HEK 293T EVs can deliver engineered IL-10 mRNA to inflammatory macrophages and activate IL-10, while in other tissues/organs, there is no significant inflammatory response in ApoE^-/-^ mice.	(Bu et al., 2021)

Intractable diseases mainly refer to chronic or rare diseases that lack clearly curable treatments. Here, we take cancers as examples. The successful distribution of medications and molecules for cancer therapy has faced numerous hurdles, including poor tissue-specific absorption, poor permeability to physiological barriers, lack of in vivo stability, and low bioavailability. Nanotechnology-based on EVs have been widely applied to solve these issues (Patra et al., 2018; Hu et al., 2021). Schindler et al. loaded DOX into BMSCs-EVs and conducted a study, which revealed that DOX exhibited reduced cardiac toxicity and decreased accumulation in the heart (Schindler et al., 2019). Wei et al. discovered that MSCs-EVs loaded with DOX reduced cytotoxicity while also inhibiting the proliferation of osteosarcoma cells (Wei et al., 2019). More significantly, EVs do not replicate or mutate, which makes them highly biosafe (Han et al., 2021; Rädler et al., 2023). As an example, Xiu et al. designed a drug delivery system RBC-EVs/gp350Etp/DOX and found that they were able to enhance toxicity to tumor cells without systemic toxicity (Xiu et al., 2022). Overall, EVs serve as carriers for the precise delivery of anticancer medications.

With increasing appreciation, besides cancers, diseases like stroke, Parkinson’s disease (PD), AD, and other conditions are being recognized. They are all closely related to the development of neuroinflammation, which occurs primarily in response to various aspects of nerve injury, infection, etc., in which glial cells play an important role as resident immune cells. For instance, Li et al. discovered that the miR-124/STAT3 pathway mediates the association between the reduction of M2 microglial-EVs and the development of glial scars following ischemic stroke in mice (Li et al., 2021). MSCs can control immune responses and act as neuroprotective agents by promoting neurogenesis, oligodendrocyte, astrocytogenesis, and angiogenesis, as demonstrated by a number of recent experimental studies in ischemic stroke (Dabrowska et al., 2019; 2019). The disruption of blood-brain barrier permeability restricts the anticipated therapeutic effect for brain illnesses. Tian et al. created a recombinant fusion protein and discovered that it dramatically increased the delivery efficacy and brain-targeting performance of human neural progenitor cell EVs (Tian et al., 2021). Additionally, it was discovered that the use of EVs from rat brain endothelial cells to load tissue plasminogen activators can enhance neurological prognosis by lowering neurological function scores (Li et al., 2021). EVs from several cell types, such as neurons and glial cells, typically play two important roles in the etiology of PD: 1. They play a crucial mediator in intercellular-syn transmission; 2. They can carry RNA (Pinnell et al., 2021). In recent years, extensive research has been conducted on the association between α-synuclein proteins and EVs derived from SH-SY5Y cells. These EVs are prospective targets for therapeutic intervention in PD because it has been discovered that they play key roles in the release and intercellular transmission of α-synuclein (Desplats et al., 2009; Alvarez-Erviti et al., 2011; Fan et al., 2019; Guo et al., 2020). Cooper et al. conducted a study investigating the therapeutic potential of siRNA in PD by examining the impact of bone marrow-derived dendritic cell EVs carrying siRNA. Their findings revealed that utilizing EVs loaded with modified α-syn siRNA effectively reduces the levels of α-syn mRNA in transgenic mice (Cooper et al., 2014). It is widely recognized that the pathology center of AD is the extracellular aggregates that form beta-amyloid polypeptides (A-β), and in previous studies, engineered EVs are potentially therapeutic in reducing A-β peptides in models of AD (Jahangard et al., 2020; Deng et al., 2021; Yu et al., 2021). Moreover, Yuyama et al. provided a description of how microglia uptake and eliminate A-β related EVs as well as A-β protein deposits (Yuyama et al., 2012). Considering the protein’s susceptibility to degradation, Sayeed et al. used a drug delivery method to encapsulate Tom 40 protein in HEK 293T cell-EVs, demonstrating the potential therapeutic efficacy in restoring mitochondrial function and addressing neurodegenerative disorders (Sayeed & Sugaya, 2022).

### 4.3. Delivery routes of physiological administration

Different delivery methods lead to various drug distributions in the body, which significantly affects the treatment outcome. Typical modes of administration include oral delivery, transdermal delivery, intravenous injection, intraperitoneal injection, and others. (Wiklander et al., 2015; Munagala et al., 2016; Zhang et al., 2020) (Table 3).

**Table 3.** Different routes for EVs-based drug delivery.

Source	Route	Advantage	Reference
Bovine milk	Oral delivery	Bovine milk EVs have increased EGGC activity.	(Luo et al., 2021)
Bovine milk	Oral delivery	Bovine milk EVs increase the solubility of drugs and make them easier to administer orally.	(Vashisht et al., 2017; Qu et al., 2022)
MSCs	Oral delivery	The concentration administered orally is half that of intravenous treatment.	(Deng et al., 2023)
Bovine milk	Oral delivery	Oral administration has a superior and more lasting hypoglycemic effect.	(Wu et al., 2022)
Colostrum powder	Oral delivery	Oral FA- EVs paclitaxel achieves higher results than traditional intravenous PAC.	(Kandimalla et al., 2021)
Human umbilical vein endothelial cells (HUVECs)	Transdermal delivery	EVs enhance collagen maturation and angiogenesis through continuous release.	(Zhao et al., 2020)
MSCs	Transdermal delivery	MSCs EVs act directly on the lesion to reduce the width of the scar and promote collagen maturation.	(Zhang et al., 2015)
Human umbilical cord MSCs (HUC-MSCs)	Transdermal delivery	By transdermal administration, EVs accelerate wound healing.	(Prausnitz and Langer, 2008)
B16BL6 cells	Intravenous injection	Bioavailability is increased, and excessive metabolism’s negative effects are diminished.	(Imai et al., 2015; Morishita et al., 2015)
HEK 293T cells	Intravenous injection	The delivered agents through intravenous injection can reach the tumor site within one hour.	(Lai et al., 2014)
MSCs	Intravenous injection	Prolongs the circulation time of the drug through the body. Drugs can be detected within two hours.	(Wei et al., 2021)
HEK 293T cells	Intravenous injection	The intravenous administration of EVs enhances circulation and lessens toxicity to other sites.	(Nguyen Cao et al., 2021)
4T1 cells	Intravenous injection	Intravenous injection improves the circulation of EVs throughout the body.	(Liu et al., 2019)
MSCs	Intranasal injection	When administered intravenously, the medication enters the target cell’s cytoplasm right away.	(Guo et al., 2019; Peng et al., 2022)
HEK 293T cells	Intranasal injection	Intranasal drug delivery bypasses the blood-brain barrier, minimizing the contact between the delivered drug and the rest of the body.	(Zhai et al., 2022)
MSCs	Intranasal injection	It can effectively cross the blood-brain barrier and improve the concentration of drugs entering the brain.	(Zhou et al., 2023)
EL4 cells	Intranasal injection	EVs-coated drugs are rapidly delivered to the brain, selectively absorbed by microglia, and subsequently induce microglia apoptosis.	(Zhuang et al., 2011)
HEK 293T cells	Intraperitoneal injection	The intraperitoneal injection can deliver the drug directly to the lesion site, reducing the spread of other sites.	(Choi et al., 2020)
Primary brain tumors	Intraperitoneal injection and intravenous injection	Both intraperitoneal injection and caudal vein injection of LCN2 can improve the transport of nanocapsules to the brain.	(Yang et al., 2022)

While oral administration is commonly preferred, the harsh gastrointestinal environment makes it challenging for medications to cross the gastrointestinal wall. As a result, the bioavailability after oral administration is significantly reduced (Tibbitt et al., 2016). Milk-derived EVs (MES) are commonly used to prepare oral formulations because they can overcome the challenges of the gastrointestinal microenvironment (Zhong et al., 2021). Early observations highlight the special benefits of MES in cargo transit through gastric shielding (Luo et al., 2021). For instance, loading curcumin on MES can enhance its stability, preventing curcumin from being broken down by human digestive enzymes, and allowing curcumin to pass across the intestinal barrier (Vashisht et al., 2017). To mitigate the toxicity of medicine to the organism, changing the route of administration is one approach. Normally, PTX is administered intravenously for the treatment of lung, ovarian, pancreatic, and cervical cancers. However, due to the negative impact of the drug on other organs and its limited solubility in water, the use of PTX-loaded bovine MES in oral administration can effectively tackle the mentioned concerns (Agrawal et al., 2017).

Transdermal administration involves directly applying medications to the skin, allowing for localized therapeutic effects on the skin, dermis, and subcutaneous fat, or enabling systemic therapeutic effects through blood capillaries (Prausnitz & Langer, 2008). Bone marrow MSCs (BMSCs) and human umbilical cord MSCs (HUC-MSCs) can elevate the proliferation and migration of keratinocytes and fibroblasts, and thus the EVs secreted from them can be used to repair wound defects, providing a new method for repairing skin wound defects (Zhang et al., 2015; Zhao et al., 2020; Zhou et al., 2023). For diabetic wound regeneration, for instance, Yuan et al. created a novel microneedle (MN) patch (GelMA/PEGDA@T + EVs MN) to promote cell migration and angiogenesis through the directed release of HUVECs-EVs and tazarotene in the deeper layers of the skin (Yuan et al., 2022). However, the problems to be considered for transdermal drug delivery are the high economic cost and the relatively high irritation to local tissues, which have to be optimized.

Intravenous injection has high bioavailability. In multiple investigations, it was discovered that 30% of EVs were still circulating after 30 min of intravenous injection (Morishita et al., 2015). The administration of drugs through intravenous delivery allows for the attainment of predictable and finely adjustable blood concentrations (Lai et al., 2014; Imai et al., 2015). In one research, Wei et al. developed a drug delivery system using MSCs-EVs loaded with CD47-EV and miR-21a. After intravenous injection, CD47-EV was still detectable in plasma after 120 min, whereas unmodified EV was identified for less than 30 min, successfully extending the time of drug circulation (Wei et al., 2021). Another research showed that a single dose of intravenous injection of EVs dramatically reduced tumor development in mice without causing systemic toxicity, indicating that intravenous EVs therapy is a secure and successful method for treating cancer (Liu et al., 2019; 2020; Nguyen Caoet al., 2021). However, intravenous injection results in the drug being predominantly enriched in the liver with limited distribution elsewhere. For clinical use, it is difficult for patients to inject themselves, and professional assistance is needed.

Nasal administration is now widely used in the treatment of numerous disorders due to the broad area of the nasal mucosa, the simplicity of absorption, the ease of administration, and the effectiveness of targeting. In 2022, Driedonks team reported for the first time the pharmacokinetics and biodistribution of expi293f-derived EVs labeled with a nano-luciferase reporter gene in a non-human primate model (rhesus macaque) and compared intravenous and nasal administrations, which revealed that intravenous EVs circulated in plasma for a longer period of time than previously reported, and that within 1 h of administration, EVs were detected in liver and spleen, while nasal administration resulted in little release in cerebrospinal fluid (Driedonks et al., 2022). Peng et al. have created an autonomous nanocarrier, PR-MSCs-EVs/PP@Cur, which after nasal administration, delivers the medication directly into the cytoplasm of the target cells, boosting the concentration of the drug (Peng et al., 2022). Furthermore, due to the blood-brain barrier’s propensity for degrading proteins, enzymes, and nucleic acids, as well as the absence of cell-specific targeting, intranasal administration of exosomal medications presents a novel therapeutic approach (Jiang et al., 2011; Zhuang et al., 2011; Guo et al., 2019; Zhai et al., 2022; Zhou et al., 2023). While nasal administration can be an effective method to bypass the blood-brain barrier, the use of nasal drops or sprays may lead to an unpleasant taste in the mouth, resulting in a negative patient experience.

Illnesses such as peritonitis, which occur in the subdiaphragmatic region, abdominal cavity, pelvic cavity, or posterior peritoneum, are most effectively treated through intraperitoneal methods. For example, intraperitoneal injection of bacterial protoplast/293T cells-EVs carrying soluble protein or bacteria can reduce abdominal abscesses and reduce inflammation (Kim et al., 2015; Choi et al., 2020).

### 4.4. Delivery routes of target cells uptake

EVs are derived from various sources, such as fibroblasts, glial cells, hematopoietic cells, etc. (Yáñez-Mó et al., 2015; Hu et al., 2016). They act as mediators of cellular communication and drug delivery, carrying combinations of ligands that can simultaneously bind to different cell surface receptors. This docking causes a signaling response, which triggers a series of events, including the translocation of membrane proteins from the vesicle to the cell membrane, fusion of the vesicle with the receptor’s cell membrane, phagocytosis of the vesicle by endocytosis, and eventual exocytosis through the vesicle-cell channel. These processes enable cells to communicate with each other without direct contact (de Curtis and Meldolesi2012). For example, APC-derived EVs facilitate antigen-specific communication between non-adjacent APCs and T cells by transferring MHC class II/peptide complexes to T cells (Arnold & Mannie, 1999).

So far, for the internalization mechanism of EVs, some reports have pointed out that it is proposed to be the fusion of the cell with the plasma membrane or endocytosis (Mulcahy et al., 2014), and there are many pathways of endocytosis, such as reticulin-mediated endocytosis, vesicle protein-mediated endocytosis, etc., and the type of cell and physiological state play a key role in the type of cellular uptake. For example, in neurons, endocytosis or phagocytosis is mainly dependent on cage proteins, whereas tumor cells are mainly dependent on endocytosis of cholesterol and lipid rafts (Montecalvo et al., 2012; Svensson et al., 2013; Yáñez-Mó et al., 2015). Additionally, early literature has demonstrated that proteins, lipids, and polysaccharides within EVs influence their uptake (Lötvall et al., 2014; Hoshino et al., 2015; Meyer et al., 2017). Tetraspanin-like proteins typically form complexes with other molecules, such as tetracyclines and integrins, which can influence their targeting behavior. For example, CD63-positive EVs have been shown to target neurons and glial cells, whereas CD63-negative EVs bind only to neuronal dendrites (Laulagnier et al., 2018). In addition, as demonstrated in earlier reports, the increased EVs uptake by melanoma cells may be attributed to the high rigidity and sphingomyelin/ganglioside GM3 (N-acetylneuraminylgalactosylglucosylceramide) content in EVs released at low pH. Hence, the combination of EVs with a pH-sensitive fusion peptide can serve as one of the methods to facilitate the endocytosis of EVs. For example, in the microenvironment of tumors, the combination of a pH-sensitive fusion peptide to promote the fusion of intracellular endosomes and EV membranes enhances cytoplasmic release of the contents of EVs into the cytoplasm (Parolini et al., 2009; Nakase & Futaki, 2015).

The routes selected for EVs depend on the doner cells and receptor cells type, so determining which uptake pathways produce high levels of functional cargo delivery is important for the subsequent successful development of EVs with therapeutic potential.

## 5. The reproducibility, safety, and scalability of EVs production and subsequent clinical translation

EVs, serving as vehicles for drug delivery, present greater promise for application compared to artificial NPs due to their targeting capabilities, surface fusion proteins, and immune evasion properties. Nevertheless, they encounter hurdles regarding the reproducibility, safety, and scalability of production, as well as their subsequent clinical translation.

Firstly, reproducibility is key for EVs production. Earlier literature reported that cell culture parameters can have a significant impact on the role of EVs production and bioactivity. Divya B. Patel et al. noted that the bioactivity of mesenchymal stem cells (MSCMs) decreased significantly when the number of generations of MSCMs exceeded the fourth generation, and furthermore, a decrease in the density of cell inoculation in culture flasks led to an increase in the production of EVs per cell in MSCs and other cell types (Patel et al., 2017). In addition, in order to conduct reproducible studies on EVs content and function, storage conditions need to have a minimal effect on EVs (Sokolova et al., 2011). Cheng et al. investigated the effects of multiple storage conditions (temperature, repeated freeze-thaw, pH) on changes in the number of EVs and cellular uptake using HEK 293T cells and the Extra PEG method, and the results revealed that relatively high temperatures and freeze-thaw cycles may affect the EVs membrane and change its properties, thus making EVs more readily available for cellular uptake and decreasing the concentration of EVs and increasing the cellular uptake of EVs under storage conditions at pH 4 (Cheng et al., 2019). In addition, the literature has been reported that adjusting cell culture parameters (pH, nutrients) as well as the use of exogenous stimulants (e.g. increasing intracellular calcium levels, thermal stimulation) can result in problems at the bottom of the yield (Taylor & Shah, 2015). For example, Jang et al. continuously extruded mononuclear cells through a filter to produce drug-loaded nanovesicles, which resulted in a 100-fold increase in yield compared to natural EVs without altering the original activity (Jang et al., 2013). Therefore, it is crucial to consider parameters such as the number of cell passages, cell inoculum density, and storage conditions during the production of therapeutic EVs.

Secondly, although the yield can be significantly improved by the methods mentioned last paragraph, the safety still needs to be further investigated. The biological origin, non-mutagenesis, and non-replicability of EVs confer a high level of safety, which has been validated both preclinically and clinically. Next, we provide a brief overview of this and discuss important considerations for the safety of EVs. In a 23-day toxicity analysis of EVs, the authors analyzed the effects of engineered and wild-type HEK293EVs on the immunoreactivity of C57BL/6 mice. These results illustrated that EVs seem to be generally well tolerated, even when used xenogenically (Zhu et al., 2017). In a phase I clinical study, researchers isolated dendritic cells from patients with advanced melanoma. These cells were then pulsed with tumor antigens. The administration of EVs was found to be well-tolerated for up to 12 months, with only one out of 15 patients experiencing an inflammatory response (Escudier et al., 2005). In another phase I clinical trial, serogroup B meningococcal outer membrane vesicle vaccines also proved effective (Sandbu, et al. 2007). However, safety studies conducted in rodents should be interpreted with caution because of fundamental differences in the activation of acute allergic reactions between rodents and large animals such as pigs. There are still many clinical trials underway to validate the safety and efficacy of EVs therapies. Because there are still some potential problems, continued research is needed to ensure that each specific application of EVs is safe.

Thirdly, the scalability of EVs also needs to be considered. Currently, small-scale EVs require stand-alone measurements on individual samples, and for the need to produce large-scale EVs, more high/medium throughput techniques are required (Erdbrügger U et al., 2021). In order to achieve the scalability of EVs analysis, automation and miniaturization of analysis in (microfluidic) devices to achieve higher throughput is now widely used. For example, Jong et al. developed a microfluidic cell culture platform that harvests antigen-modified EVs in a single workflow, allowing for the rapid, real-time generation of therapeutic EVs (Jong et al., 2017). But it’s highly dependent details in the protocols and settings (Staubach et al., 2021). Therefore, it is highly recommended to truly understand the process, identify key process parameters, and recognize any changes in the production process as early as possible, not just at the end of the process.

Last but not least, the inefficiency of delivering drugs to target tissues presents a challenge in clinical translation. For example, blood-derived EVs are difficult to enrich into focal areas without modifying effects (Qi, et al. 2016). Most cells secrete EVs with limited tropism for specific cell types, limiting the efficacy of the drug (Barile & Vassalli 2017). Recent studies have focused on addressing this problem for EVs by exploiting the inherent ability of EVs-encapsulated organisms, surface-targeted modifications, and chemical reagents. The study conducted by Perets et al. demonstrated the crucial role of inflammation in attracting and guiding the migration of EVs derived from mesenchymal stem cells (MSCs) toward various brain pathologies, such as stroke, autism, PD, and AD. After 96 h, MSC EVs remained and increased accumulation in certain regions, which was mainly attributed to the inherent homing ability with MSCs. This provides a basis for clinically targeted diagnosis and therapy (Perets et al., 2019).

Currently, the field of EVs is still a blue ocean, both in the clinical field of application and in the field of medical esthetics are gradually improving. In the United States, EVs therapy is approved and regulated by the FDA. In general, EVs used for the treatment of diseases, need to be regulated as drugs and biological products in accordance with the Public Health Service Act and the Federal Food Drug and Cosmetic Act, and are subject to pre-market review and approval requirements. Currently, there is no regulatory approval for EVs drugs in any country in the world, and rigorous scientific research and effective regulation in this area are still needed in the future to truly realize the clinical value of EVs.

## 6. Concluding remarks and perspective

In the past decades, tremendous EVs from different origins have been studied for the development of novel targeted drug delivery systems. They have agent-loaded space and modifiable areas, enabling the realization of loads of many therapeutic ingredients and imaging agents, and the integration of targeting ligands, without affecting their initial advantages. The unique advantages of different EVs, make them promising candidates for target-selective delivery, especially ligand-mediated targeting. This review primarily focuses on the current state of EVs-based strategies in targeted drug delivery, describing the correlated production, modification, and examples.

First of all, owing to the broad sources of EVs, EVs are regarded as cost-effective therapeutic carriers. Almost all cells secrete EVs, which harbor a plethora of different biomolecules for transporting across cells. Different EVs show target specificities that can deliver cargoes to parental cells expressing complementary receptors or molecules. As reported, they can be delivered as Trojan horses, bringing hope to intractable diseases (Patras et al., 2022; Klimova et al., 2023). Given their superiority, the improvement of productivity of EVs is highly demanded for further applications. Therefore, we concluded the mass production of EVs.

Second, since EVs have inspired the development of active drug components or drug delivery systems, in-depth comprehension of drug loading and active targeting methods could contribute to the future design of EVs-based systems. Existing modification methods are classified including pre-modification, per-modification, and post-modification, covering all modification methods. These modification methods have expanded the scope of EVs-based engineering beyond natural receptor targeting systems and aim to integrate complicated modules granting multiple functionalities simultaneously, enabling the enhancement of the specific recognition and precision targeting for further improving therapeutic potential. Researchers can refer to these methods to explore the most efficient and suitable modification methods in their studies.

Third, indeed, the fully understand of the roles of EVs remain a daunting challenge. The current state of EVs-based strategies in targeted drug delivery is still in its infancy. These strategies require combinations of known features. Though the nature or modification of EVs has realized the expected function, the potential of which needs more exploitation. We can only list limited examples from the aspects of drug models, disease models, delivery routes of physiological administration, and delivery routes of target cells uptake. It is believed that more progress will be achieved as study and discovery proceed further.

Finally, to drive next-generation therapeutics, the pros and cons of EVs should be regarded dialectically. EVs-based strategies have achieved great advancement in targeted drug delivery, nevertheless, the safety issues remain to be solved. Because there exist many unknowns in EVs compared to traditional synthetic drug delivery systems which have traceable individual components. Here is a need to use multiple approaches to assess the diverse composition of EVs, the associated risk, and safety. In fact, the diverse composition of EVs increases the difficulty of research. To our knowledge, the biggest difficulty of EV-based targeted drug delivery comes from the abstruse of EVs. The biogenesis as well as the uptake and transportation mechanisms lead to the complexities of EV-based targeted drug delivery. In addition, the absence of a recognized procedure in the production process of such delivery systems is also a problematic issue. The rigor and reproducibility of EV production should be addressed.

In conclusion, with the continuous advancement of technology and in-depth research, it is hoped that above mentioned issues will be solved gradually. EVs can be positioned as potential targeted drug delivery systems for treating various diseases with medical unmet needs in the future.
